# Lecture on Æthusa

**Published:** 1885-07

**Authors:** J. T. Kent

**Affiliations:** St. Louis


### LECTURE ON ÆTHUSA.

PROFESSOR J. T. KENT, A. M., M. D., ST. LOUIS.

The most important feature of Æthusa is its vomiting, after which (and sometimes preceding) come the convulsions—vomiting of a substance looking like "smearcase;" there is a diarrhœa of undigested milk—green, slimy, sometimes bloody; there may be convulsions attending this vomiting, but they most generally come after; eyes turned downward and thumbs convulsed across the palm of the hand, or sometimes they are turned back. After the child has been vomiting, great prostration and drowsiness come on; it wakes up from sleep, partly refreshed, and calls for the breast and nurses to repletion, and then vomits the milk before it has coagulated; a peculiar prostration and sinking come on after this continual vomiting; blue, sunken line of the upper lip, running to the nose—linea nasalis; body may be cold and the head hot (also Arnica), with cold sweat all over, and then follow convulsions; child lies unconscious; dilated pupils, staring eyes, confused idiocy, with great sadness when alone; upper lip white to the nose, which occurs after exhaustion, and diarrhœa; aphthæ in the mouth and on the tongue, with the peculiar vomiting, followed by sleep; sudden vomiting of frothy matter; regurgitation of food an hour after eating; painful contractions of the stomach—so severe as to prevent vomiting; the patient will strain to vomit, as if he would tear his stomach open (the chronic regurgitation of food or fluids two or three hours after eating—Sulphur); epileptic spasms, with clenched thumbs; red face; eyes turned downward and dilated, staring pupils (something like Cuprum, which compare); dozing of child after vomiting spell; ecchymoses all over; red spots on skin, like water-blisters, from lying on bed.

